# TREATMENT OF INFERIOR POLE FRACTURE OF THE PATELLA IN ADULTS: SYNTHESIS OF CLINICAL EVIDENCE

**DOI:** 10.1590/1413-785220263402e298167

**Published:** 2026-05-22

**Authors:** João Pedro de Melo, Stephanie Brandão, Diego David Vilte Martinez, Alessandra Masi, Carlos Gorios

**Affiliations:** 1 Hospital Geral de Carapicuíba, Carapicuíba, Sao Paulo, SP, Brazil.

**Keywords:** Orthopedics, Traumatology, Knee, Surgery, Fractures, Bone, Patella, Ortopedia, Traumatologia, Joelho, Cirurgia, Fraturas, Patela

## Abstract

Fractures of the Inferior pole patellar fracture (IPPF) affect the distal end of the bone, where the patellar tendon inserts, and are more common in young, active adults due to direct trauma or intense muscle contractions. This region, which is structurally more fragile and subjected to high tensile loads, becomes vulnerable to fractures that compromise the knee extensor mechanism, resulting in pain, functional limitation, and loss of mobility. Treatment can be conservative or surgical, depending on the degree of displacement and the integrity of the extensor apparatus, and involves various techniques, including minimally invasive procedures. Given the clinical relevance and controversy regarding optimal management, it is necessary to synthesize the available evidence to support therapeutic decisions and optimize functional outcomes. To review the literature on the treatment of IPPF in adults. The search was performed in the PUBMED database using the following search strategy: *inferior[title] AND pole[title] AND patella[title] AND fracture[title]*, with a time cut-off of five years.

**Results:**

Initially, 22 studies were identified that met the previously established search strategy. After reading the titles and abstracts, six studies were excluded, leaving 16 articles that constituted the sample for this review. The studies demonstrated stable fixation techniques for IPPF that favored bone consolidation and early functional recovery, but the choice of the ideal method still depends on the type of IPPF, the surgeon's experience, and the reduction of complications such as reoperations, in addition to the presence of a low patella. ***Level of Evidence IV, Case Series.***

## RESUMO

As fraturas do polo inferior da patela (FPIP) acometem a extremidade distal do osso, onde se insere o tendão patelar, sendo mais comuns em adultos jovens e ativos devido a traumas diretos ou contrações musculares intensas. Essa região, estruturalmente mais frágil e submetida a altas cargas de tração, torna-se vulnerável a fraturas que comprometem o mecanismo extensor do joelho, resultando em dor, limitação funcional e perda de mobilidade. O tratamento pode ser conservador ou cirúrgico, conforme o grau de deslocamento e a integridade do aparelho extensor, e envolve técnicas variadas, incluindo procedimentos minimamente invasivos. Diante da relevância clínica e da controvérsia quanto ao manejo ideal, torna-se necessário sintetizar as evidências disponíveis para embasar decisões terapêuticas e otimizar os desfechos funcionais. Revisar a literatura acerca do tratamento da FPIP em adultos. A pesquisa foi realizada na base de dados PUBMED, utilizando a seguinte estratégia de busca: inferior[title] AND pole[title] AND patella[title] AND fracture[title], com um corte temporal de cinco anos.

**Resultados:**

Inicialmente foram identificados 22 estudos que atenderam a estratégia de busca previamente estabelecida. Após a leitura dos títulos e resumos, 6 trabalhos foram excluídos, restando 16 artigos que efetivarem constituíram a amostra da presente revisão. Os estudos demonstraram técnicas de fixação estável para FPIP que favorecessem a consolidação óssea e a recuperação funcional precoce, mas a escolha do método ideal ainda depende do tipo de FPIP, da experiência do cirurgião e da redução de complicações como reoperações, além da presença de patela baixa. ***Nível de Evidência IV, Série de Casos.***

## INTRODUCTION

The inferior pole of the patella corresponds to the distal portion of the patellar bone, where the patellar tendon inserts, forming part of the knee extensor complex.^1^ Structurally, this region has less bone thickness and is subjected to high tensile loads during activities that involve flexion-extension under resistance.^2^ This configuration makes the inferior pole susceptible to fractures, especially in situations of direct trauma or violent contraction of the quadriceps.^3^

The inferior pole patellar fracture (IPPF) consist of bone injuries that affect the distal end of the patella, often resulting in separation of the tendon insertion fragment.^4^ Etiologically, they are associated with direct traumatic mechanisms to the knee or traction caused by muscle contractions, such as falls with the knee flexed.^5^ These fractures compromise the extensor mechanism, leading to the inability to actively extend the leg, pain, functional limitation, and loss of mobility, impacting the patient's quality of life.^6^

Patellar fractures account for about 1% of all fractures, with IPPF responsible for a significant fraction of these cases, especially among young adults and active individuals.^6^ They are more prevalent in adult males aged between 20 and 50 years due to greater exposure to physical activities and direct trauma. In the elderly, they also occur associated with osteoporosis and bone fragility.^2^ The nature of the trauma determines the fracture pattern, with IPPF classified as transverse fractures with detachment of the patellar tendon.^2,6^

The treatment of IPPF can be conservative or surgical, depending on the degree of displacement of the fragment and the integrity of the extensor apparatus.^2^ In cases without detachment or instability, immobilization and physical therapy may be indicated.^5^ However, in unstable fractures or with loss of the extensor mechanism, surgical intervention is generally necessary.^4^ Techniques include resection of the fractured fragment with reinsertion of the patellar tendon, fixation with wires or anchors, and the use of biocompatible materials and minimally invasive techniques for anatomical and functional restoration.^2,4^

Given the importance of the patella and the impact of fractures on gait, knee extension capacity, and patient independence, it becomes essential to understand the most effective approaches. Although relatively common, inferior pole patellar fracture still generate controversies regarding treatment, varying according to the technique used, outcomes, and rehabilitation time. Thus, this work is justified by the need to synthesize the available evidence, promoting technical-scientific support for decision-making and improvement of outcomes in affected patients.

## OBJECTIVE

Review the literature on the treatment of IPPF in adults.

## METHOD

This study was conducted based on the integrative literature review method, focusing on the synthesis of evidence. The research was conducted in the PUBMED database, using the following search strategy: *inferior[title] AND pole[title] AND patella[title] AND fracture[title]*, with a five-year cutoff. All types of articles were included for evaluation, with no restrictions regarding the methodological model. This review followed the methodological steps described by Souza et al.^7^ which include: (a) formulation of the guiding question; (b) survey of published studies; (c) preliminary selection of articles for analysis; (d) critical evaluation of studies by experts; (e) discussion of the results of the evaluated articles; and (f) synthesis of the convergences and divergences among the analyzed works. The guiding question of this review was: "what clinical evidence is available on the most effective treatment methods for inferior pole patellar fracture in adults?".

## RESULTS

Initially, 22 studies were identified that met the previously established search criteria. After reading the titles and abstracts, six articles were excluded for not addressing the treatment of IPPF in adults. The remaining 16 articles were read in full, summarized, and presented in the next section in chronological order of publication year. Table 1 presents a summary of the information related to the reviewed works.

**Table 1:** Summary of the information related to the works.

Authors	Title	Type of Study	No. of patients	Conclusion
He et al.^8^	*Novel Rim Plating Technique for Treatment of the Inferior Pole Fracture of the Patella*	Prospective observational study	4	The technique proved effective, with bone consolidation in 6–9 weeks and excellent functional results without complications.
Chang et al.^5^	*Fracture of the inferior pole of the patella: tension band wiring versus transosseous reattachment*	Retrospective cohort study	55	The tension band wiring (TBW) and transosseous reattachment (TOR) were effective, but displacement >30 mm increased the risk of radiological loss of reduction; 60% of cases with TBW required implant removal.
Wang et al.^9^	*A case-control study of two internal fixation methods in the treatment of comminuted fracture of inferior pole of patella*	Retrospective comparative study	60	The group that used the Patellar Concentrator in Memory Alloy (NiTi PC) had a better Böstman score, fewer complications, and better functional recovery, being an effective alternative for comminuted IPPF.
Lu et al.^10^	*"Fishing net" suture augmenting tension-band wiring fixation in the treatment of inferior pole fracture of the patella*	Clinical study	37	Fixation with "fishing net" demonstrated better stability and fewer failures, being a viable alternative to TBW.
Kim et al.^11^	*Suture anchor fixation of comminuted inferior pole patella fracture-novel technique: suture bridge anchor fixation technique*	Retrospective clinical study	22	All patients achieved bone consolidation and good range of motion (ROM); functional scores were satisfactory, indicating good results from the technique.
Lin et al.^12^	*Treatment of comminuted fracture of inferior pole of patella with locking suspension and vertical fixation with three steel wires*	Prospective clinical study	23	The technique showed reliable fixation, high consolidation rate, rapid rehabilitation, and satisfactory clinical effect.
Gao et al.^13^	*Treatment of avulsion fracture of inferior pole of patella with improved angle of anchor and double pulley technique*	Retrospective study	22	Technique of easy execution with satisfactory curative effect and good functional recovery of the knee joint.
Pu et al.^14^	*Treatment of inferior pole fracture of the patella with tension-free external immobilization*	Retrospective cohort study	11	Viable treatment, allowing early functional exercise with satisfactory clinical results.
Ma et al.^15^	*Treating Inferior Pole Fracture of Patella with Hand Plating System: First Clinical Results*	Retrospective cohort study	30	Secure fixation with excellent bone consolidation rate, adequate functional recovery, and absence of relevant complications.
Liu et al.^16^	*Tension-band wiring through a single cannulated screw combined with suture anchors to treat inferior pole fracture of the patella*	Retrospective cohort study	22	Technique provides good stability with low surgical complexity and satisfactory clinical results.
Zhou et al.^17^	*Treatment of inferior pole patella fracture using Krackow suturing combined with the suture bridge technique*	Retrospective cohort study	18	The combined technique showed stable fixation and good clinical results, with good/excellent functional recovery in 100% of patients.
Kim et al.^18^	*Comparison of Fixation Methods Between Transosseous Pull-Out Suture and Separate Vertical Wiring for Inferior Pole Fracture of Patella: A Systematic Review and Meta-Analysis*	Systematic review and meta-analysis	16 studies (274 patellas)	Both techniques showed satisfactory and similar results; supplemental fixation reduced bone complications.
Nair et al.^19^	*Inferior Pole of the Patella Fracture Fixed by Percutaneous Approach: A Case Report*	Case report	1	Percutaneous fixation was effective and minimally invasive, with good functional results in six weeks.
Solunkhe et al.^20^	*Comparative Study of Inferior Pole of Patella Fracture Treated with Transosseous Technique using Modified Krackow's Technique Versus Traditional Anterior Tension Band Wiring*	Comparative Study	20	KT proved to be a viable alternative to TBW, with comparable results in ADM, radiology, and WOMAC score.
Murase et al.^21^	*Fracture of the patella involving the inferior pole is associated with postoperative patella baja - A retrospective multicenter study*	Retrospective cohort study	251	Inferior pole patellar increased from 14.3% to 19.5% in six months; IPPF on computed tomography was identified as a risk factor, but there was no difference in clinical outcomes between the groups.
Yan et al.^22^	*Krackow suturing combined with the suture-bridge technique versus Kirschner-wire tension band combined with patellar cerclage for the treatment of inferior pole patella fracture: a retrospective comparative study*	Retrospective comparative study	47	Both techniques were effective, but the suture bridge technique had fewer complications, lower cost, and a lower reoperation rate.

Source: Data collected by the authors.

## DISCUSSION

He et al.^8^ presented a fixation technique for the treatment of IPPF. To this end, they conducted a prospective observational study of consecutive cases of IPPF that were treated at a single clinical center between January 2018 and June 2019. The patients included three men and one woman, with an average age of 47 years (42-59 years). All patients were treated with a fixation technique using a plate that preserved the inferior pole of the patella. During surgery, a 2.4 mm locking compression plate was contoured to fit the inferior arch of the patella. After reduction, the plate was fixed to the proximal fragment using locking screws, against the traction of the patellar tendon. The plate surrounded and compressed the inferior pole fragments, functioning as a compression and locking construct. When necessary, an additional anterior tension band or a mini-plate could be used to prevent anterior displacement of the inferior pole fragments. Under this fixation, knee movement and weight-bearing were encouraged postoperatively. Patients were followed up monthly for 12 months after surgery. The time to achieve a 90° movement without pain, full range of motion (ROM), and consolidation was recorded. Complications were monitored, including infection, loss of reduction, fixation failure, anterior knee pain, and soft tissue irritation. The modified Cincinnati knee classification system was used for functional assessment, and the average operative time was 58.8 minutes (52-63 minutes). The average blood loss was 59.8 mL (45-71 mL), and for all patients, the ROM of 90° without pain was restored in two to four weeks, with full ROM in 8 to 11 weeks. All patients achieved bone consolidation in six to nine weeks, with no displacement of fragments or breakage of the implant. No patient complained of pain in the anterior region of the knee or soft tissue irritation. The modified Cincinnati score at the 12-month follow-up showed excellent results in all patients. For the authors, the edge plate technique could be a viable option for the treatment of IPPF. Chang and colleagues,^5^ compared clinical and radiological outcomes after IPPF fixation with tension band wiring (TBW) and transosseous refixation (TOR) without excision of the bone fragment and determined the risk factors for postoperative loss of reduction. For this retrospective cohort study, patients with IPPF were recruited between January 2010 and December 2017. Patients were grouped according to the fixation method (TBW or TOR), and demographic data, clinical outcomes, and postoperative Insall-Salvati (IS) index were analyzed. Then, patients were grouped according to loss of reduction, potential risk factors for loss of reduction were identified, and odds ratios were calculated. The study included 55 patients; 30 patients treated with TBW and 25 with TOR. Clinical failures occurred in two patients in the TBW group (7%) and three in the TOR group (12%). The rate of loss of reduction was significantly higher in the TOR group, while implant removal was more common in the TBW group. Low patella was observed after surgery in the TOR group, but the IS ratios of the two groups were similar after three months. Fracture displacement of more than 30 mm was the only independent risk factor for postoperative loss of reduction. According to the authors, in the treatment of IPPF, both TBW and TOR were effective and had a low failure rate. In 60% of patients undergoing TBW, however, additional surgery was required to remove the implants. Low patella occurred after TOR, but patellar height was similar to that of the TBW group after three months. The authors commented that surgeons should be aware of the risk of postoperative loss of reduction, especially when the fracture displacement is greater than 30 mm.

Wang et al.^9^ compared the effects of tension band combined with cerclage and patellar concentrator fixation in memory alloy in the treatment of comminuted IPPF. To this end, from July 2015 to July 2019, 60 patients with patellar fractures were treated and divided into two groups according to different surgical methods. In group A, 30 patients were fixed with a patellar concentrator in memory alloy (NiTi PC), 17 men and 13 women, aged between 20 and 71 years (39.4 ± 9.9), including 19 cases of falls, nine traffic accidents, and two sports injuries. The time between injury and surgery was 10 to 75 hours (33.1 ± 7.8); thirty cases in group B were fixed with tension band and cerclage, patients aged between 21 and 76 years (38.6 ± 10.2), including 17 falls, 12 traffic accidents, and one crush injury. The time between injury and surgery was 10 to 91 hours (34.5 ± 9.1). The effects of the two groups were compared. All patients were followed up for 9 to 30 months, and there was no significant difference in intraoperative bleeding, operative time, follow-up time, and consolidation time between the groups. Six months later, according to the Böstman function score of the knee joint: In 30 cases in group A, the total score was 28.6 ± 4.7, of which 26 were excellent and 4 were good. The score of 30 cases in group B was 25.5 ± 4.4, of which 20 were excellent, eight were good, and two were poor. There were differences in the Böstman score and the assessment of the healing effect. The score of group A was better than that of group B, with one case in this group having the Kirschner wire removed, two presenting joint stiffness, and three presenting irritation of the internal fixation. For the authors, the memory patellar concentrator was robust and reliable in the treatment of comminuted IPPF. However, rehabilitation exercises may be necessary after surgery, with good recovery of function and ROM, as well as fewer complications.

Lu and colleagues,^10^ evaluated the biomechanical resistance and outcomes of a new fixation procedure for IPPF with "fishing net" suture. To this end, four finite element models, fixation with modified TBW wire, with anchor suture, with basket plate, and with "fishing net" suture were constructed to compare the effectiveness of the latter with the other three methods during IPPF fixation. From January 2018 to February 2019, 17 patients treated with "fishing net" suture fixation (FNS) were compared with 20 patients treated with TBW in a database, and both groups were evaluated using the Cincinnati knee classification system. The biomechanical assessment showed that the values of proximal patellar relative displacement, measured by three pairs of points on both sides of the fracture line, were lowest using FNS fixation, while TBW and basket plate fixation showed similar, less desirable stability levels than the "fishing net" method. Regarding the outcomes, 17 (100%) patients had excellent or good results, compared to three failures in internal fixation in the TBW group. According to the authors, the biomechanical results suggested that the "fishing net" fixation was a candidate for IPPF fixation.

Kim et al.^11^ evaluated the results of bridge suture anchor fixation for comminuted IPPF. To this end, from March 2012 to December 2018, 22 patients were evaluated. 21 patients with comminuted IPPF and 1 with avulsion fracture of the lower periosteal cuff. The results were evaluated, including the SF-36 score, the *Knee Injury and Osteoarthritis Outcome Score* (KOOS), and the postoperative ROM. In all patients, fixation was performed with a bridge suture anchor, and in two patients, a tension band with K-wire was added for fixation of large fragments. Additionally, consolidation and patellar height were evaluated using the Insall-Salvati ratio and its complications. The mean age was 46 ± 20 (15-82) years, and the mean follow-up period was 25 ± 18 (11-74) months. In all patients, consolidation was achieved at four months. At the final follow-up, the average score on the SF-36 was 72 ± 15 (30-91) points, and the KOOS score was 66.7 ± 16 (43-97). The ROM was 134 ± 5 (125-140) degrees, and as a complication, one patient developed a wound infection and subsequent osteomyelitis of the IPPF. Compared to the normal knee, the Insall-Salvati index of the injured knee is, on average, 0.73, and this index less than 0.8 indicates a low patella. According to the authors, in IPPF, fixation with a bridge anchor demonstrated good consolidation and satisfactory results in short-term follow-up, which may be a satisfactory treatment option. Furthermore, they also commented that although fixation with a bridge suture anchor in these fractures caused a decrease in the Insall-Salvati ratio (patellar height), there was no development of patellofemoral pain or limitation of ROM.

Lin and colleagues,^12^ explored the application of Lockge suspension combined with vertical fixation with steel wires in IPPF. To this end, from August 2016 to May 2019, 23 patients with comminuted IPPF, including 14 men and nine women, were treated with Lockge suspension combined with fixation with steel wires. The age of the patients ranged from 34 to 68 years (55.0 ± 1.2), and one year after the operation, pain and function were evaluated using a visual analog scale (VAS) for pain and knee ROM, with efficacy assessed by the Lysholm score. All 23 patients were followed for 12 to 14 months, with an average of (13.0 ± 0.5) months. One patient had skin irritation caused by the tail of the steel wire, and the rest had no complications, such as incision infection, loosening of internal fixation, or fracture displacement. The fractures of 23 patients healed, and the healing time averaged (12.0 ± 1.1) weeks. The VAS score decreased from 7.96 ± 0.93 before the operation to 0.83 ± 0.65 one year after the operation. The range of motion of the knee increased from (20.30 ± 8.69) ° to (127.39 ± 6.55) ° in 1 year. The Lysholm score of the knee increased from 18.48 ± 4.00 to 96.09 ± 4.91 one year after the operation. According to the authors, the treatment of comminuted IPPF with blocked suspension combined with vertical fixation using steel wires showed reliable fixation and a high rate of fracture consolidation. Moreover, the technique met the requirements for rapid rehabilitation and functional exercise, and the clinical effect was satisfactory.

Gao et al.^13^ evaluated the positioning angle of the anchoring pin and the healing effect of the double pulley technology in the treatment of IPPF due to extreme avulsion. To this end, from December 2015 to December 2018, a total of 22 patients (10 men and 12 women) with IPPF due to avulsion were analyzed retrospectively. The average age was 44.00 ± 15.24 years (ranging from 19 to 70 years), and all patients were treated with a modified anchoring angle and double pulley technique. The range of motion and the Böstman score were used to assess the functional recovery of the knee joint. All 22 cases were followed up for an average of 30.86 ± 8.00 weeks (18 to 46 weeks). At the last follow-up, the range of motion of the affected knee was 130.82 ± 4.69° and of the contralateral knee was 133.23 ± 3.15°, with no significant difference between the two groups. The average Böstman score was 28.45 ± 1.41, with 18 cases showing excellent results and four cases showing good results. According to the authors, the improvement of the positioning angle of the anchor and the double pulley technique for the treatment of FPIP due to avulsion were easy to perform, with satisfactory healing effect and good recovery of knee function. Pu and colleagues,^14^ proposed a new treatment method for IPPF with non-tension external immobilization.

To this end, the clinical data of 11 patients with IPPF treated with non-tension external immobilization between May 2016 and June 2019 were analyzed. The study subjects were six male patients and five female patients, with ages ranging from 39.0 ± 12.8 years (age range of 18 to 53 years). The average age was 44.00 ± 15.24 years (ranging from 19 to 70 years), and all patients were treated with a modified anchoring angle and double pulley technique. The preoperative range of motion of the knee was 22.0 ± 7.5° (10-30°) and the time until the operation was 4.5 ± 1.3 days (3 to 7 days). The preoperative range of motion of the knee was 22.0 ± 7.5° (10-30°) and the time until the operation was 4.5 ± 1.3 days (3 to 7 days). The indices related to the operation were recorded, and the knee function was evaluated using the Böstman score. All surgeries were successful, with an operative time of 56.4 ± 8.4 minutes (45 to 70 minutes). The average follow-up time was 20.4 ± 7.6 months (12-36 months), the duration of fracture consolidation was 8.9 ± 1.5 weeks (7-12 weeks), and the time for removal of the immobilization device was 10.4 ± 0.9 weeks (9-12 weeks). At the last follow-up, the range of motion showed no significant difference between the affected knee (129.7 ± 3.3°, range of 125-135°) and the unaffected knee (130.8 ± 3.8°, range of 126-137°). Additionally, the Böstman score for the knee was 29.2 ± 1.0 points (27-30 points), including 10 excellent cases (90.9%) and one good case (9.1%). For the authors, external non-tension immobilization was a viable treatment for IPPF, potentially aiding in early functional exercise and achieving a satisfactory clinical effect.

Ma et al.^15^ presented the Hand Plate System (HPS), a new surgical technique for IPPF, and reported the results after the application of the procedure. To this end, they outlined a retrospective cohort study that took place between July 2017 and December 2018. Thirty patients diagnosed with IPPF without additional injuries were observed, and after X-ray and 3D computed tomography examinations, all patients underwent open reduction and internal fixation using HPS, with or without supplemental stabilization with a cannulated screw and fixation screw. Bone consolidation time, range of motion, Böstman score, VAS, and complications were measured as clinical outcomes at a minimum of 12 months of follow-up. All surgeries occurred without issues, with an average operative time of 76.2 ± 15.3 minutes. Bone consolidation was achieved in all cases, averaging 9.5 ± 1.4 weeks after surgery. There was no loss of reduction, failure of the fixator, or surgical removal of the implant during follow-up. The average range of motion after one year post-operation was 0° to 123.3°, and the average Böstman score at the last follow-up was 26.8 ± 2.1, with a satisfaction rate of 100%. The sensation of pain while walking, measured by the VAS, averaged 0.9 ± 1.3, and there were no complications except for one case of poor incision healing, which eventually healed after surgical debridement. For the authors, the EHP proved to be a safe fixation method.

Liu and collaborators,^16^ evaluated the feasibility and results of tension band fixation using a single cannulated screw with two suture anchors in the treatment of IPPF. To this end, between September 2018 and September 2021, 22 patients with an average age of 55 years who suffered IPPF were included and treated with tension band fixation using a single cannulated screw combined with two suture anchors. Radiographs were performed to observe the bone consolidation time, and the duration of each surgery was recorded to reflect the complexity of the treatment. Functional measurements were taken, including range of motion, Böstman scale, and KOOS. Complications, including fixation failure, incision infection, loss of reduction, and malunion, were evaluated. All patients were followed for an average of 17 months (12-25 months), with an average consolidation time of 11 weeks (8-12 weeks). At the final follow-up, the average range of motion was 136° (range: 115°-140°), the KOOS was 85 (range: 68-100), and the Böstman score was 28 (range: 20-30); these results were classified as excellent in 17 cases and good in five cases, with no cases of poor outcomes. Loss of reduction occurred in one case, while no cases of incision infection, fixation failure, or malunion were observed. According to the authors, for IPPF, fixation with a tension band through a single cannulated screw with suture anchors could provide sufficient fixation stability to achieve a satisfactory clinical outcome with reduced surgical complexity. Zhou et al.^17^ evaluated the feasibility and clinical effect of Krackow suture combined with the bridge suture technique for the treatment of acute IPPF. To this end, 18 patients with acute IPPF who received treatment with Krackow suture combined with the bridge suture technique between January 2019 and March 2020 were reviewed. The average age was 44.00 ± 15.24 years (ranging from 19 to 70 years), and all patients were treated with a modified anchoring angle and double pulley technique. There were 10 men and eight women, with an average age of 50.1 years (range from 24 to 69 years). Radiographic examinations were performed to assess fracture consolidation and the IS index. The clinical effect was measured by the ROM of the knee joint and the Böstman scale. Patients were followed for 13 to 26 months, with an average follow-up period of 19.6 months. The X-ray indicated that fracture consolidation occurred in all patients on average 10.1 weeks after surgery (range from eight to 14 weeks). The average IS index immediately after surgery and at the final follow-up was 0.98 ± 0.07 and 0.90 ± 0.22, respectively. At the last follow-up, the average flexion and extension range of the knee joint were 135.8° ± 8.8° and −2.8° ± 3.9°, respectively, and the average Böstman scale was 28.9 ± 1.1 points. Functional recovery was excellent in 17 patients and good in one patient, resulting in an overall good/excellent recovery rate of 100%. According to the authors, the results indicated that Krackow suture combined with the bridge suture technique could achieve stable fixation of acute IPPF, providing good clinical outcomes, making it worthy of clinical application.

Kim and colleagues,^18^ compared through a systematic review the fixation methods with Krackow transosseous suture (KT) and with separate vertical wire fixation (VW) in IPPF and assessed whether supplemental fixation affected bone consolidation. To this end, the MEDLINE, Embase, and Cochrane databases were searched from their inception until January 15, 2023. The keywords were "*patella inferior pole fracture*", "*patella distal pole fracture*", "*transosseous*", "*pull-out suture*", "*reattachment*", and "*vertical wiring*". All clinical studies that described KT or VW techniques for IPPF and reported complications related to bone consolidation were included. The meta-analysis included 16 studies with 274 patellas with demographic data, surgical techniques, clinical outcomes, and recorded complication rates. The Methodological Index for Non-Randomized Studies criteria were used to assess the quality of the publications. The techniques used for the analysis were random effects models and meta-regression. The meta-analytic estimate of complications related to bone consolidation was 3.8% for KT or VW techniques in fractures of the lower pole of the patella. The rates of complications related to bone consolidation did not differ significantly between the two techniques (KT, 5.7%; VW, 3.0%). Meanwhile, fixation with supplementation was significantly associated with a reduction in rates of complications related to bone consolidation. For the authors, the fixation of IPPF using KT or VW techniques provided satisfactory and similar clinical results, with minimal complications related to bone consolidation. For them, supplemental fixation would have a positive impact on reducing complications related to bone consolidation in IPPF after KT and VW techniques.

Nair et al.^19^ reported the case of a treatment for IPPF using a percutaneous approach. The case involved a 70-year-old female patient who presented significant pain and swelling in the knee after a fall. The radiographic examination revealed a displaced fracture of the lower pole of the patella, along with a fracture of the ipsilateral tibial plateau. Surgical intervention was deemed necessary due to the extent of the displacement and the potential compromise of knee function. A percutaneous technique was employed for the reduction and fixation of the fracture using cannulated screws under fluoroscopic guidance. Post-operative rehabilitation focused on early mobilization and strengthening exercises, and at a six-week follow-up, the patient demonstrated satisfactory clinical results with restoration of knee function and minimal residual symptoms. For the authors, this case highlighted the effectiveness of percutaneous fixation in the treatment of IPPF, offering a minimally invasive approach with favorable functional outcomes.

Solunkhe and colleagues,^20^ evaluated 20 patients divided into two groups of 10 each: Group A, composed of patients operated on with TBW and Group B, with patients operated on with Krackow KT. Both groups underwent the same physiotherapy in the post-operative period and the results were compared. The radiological results and the WOMAC score (*Western Ontario and McMaster Universities Osteoarthritis Index*) of the patients were comparable in both groups. The return to total ADM was also similar in both groups, with similar physiotherapy administered in both. For the authors, this demonstrated that, with careful patient selection, KT of IPPF can be used as an alternative to TBW.

Murase et al.^21^ aimed to clarify the incidence of low patella after IPPF surgery, as well as the clinical outcomes associated with and without the presence of low patella. Additionally, the researchers also clarified the potential correlation between the detection of IPPF on computed tomography and the occurrence of low patella. To this end, they conducted a multicenter retrospective study involving 251 patients who underwent surgical treatment for IPPF. The patients were divided into the low patella group (PB; n = 49) and the normal patella group (PN; n = 202). The collected data included demographic data, radiographic findings, surgical details, and postoperative complications. Logistic regression analyses were used to identify risk factors for low patella. Immediately after surgery, 36 (14.3%) patients presented with low patella, a number that increased to 49 cases (19.5%) at six months postoperatively. There was no statistically significant difference in demographic data, surgical details, clinical outcomes, and complications between the PB and PN groups. Meanwhile, in the radiographic evaluation, the prevalence of IPPF on computed tomography in the low patella group was significantly higher than in the normal patella group. By logistic regression analysis, IPPF on computed tomography was identified as an independent risk factor for low patella. For the authors, in patients with patellar fractures, the incidence of low patella increased from 14.3% immediately after surgery to 19.5% at the six-month assessment. No significant differences were observed in clinical outcomes between the low patella group and the normal group. Additionally, the identification of IPPF on computed tomography emerged as a predictive factor for low patella.

Finally, Yan and collaborators,^22^ conducted a retrospective study of 47 patients with IPPF who underwent fixation procedures at a single clinical center between January 2019 and May 2022, of which 25 received Krackow suture combined with the bridge suture technique (Group 1) and 22 received a tension band with Kirschner wire combined with patellar cerclage (Group 2). The operative time, reoperation rate, Böstman score, knee ROM, fracture consolidation time, IS index, complications, and hospital costs were compared between the two groups. The mean follow-up period was 23.1 ± 5.8 months, and the rates of complications and reoperations in Group 2 were significantly higher than in Group 1. While Group 1 had lower hospital expenses than Group 2, no significant differences were found regarding the Böstman score, knee ROM, IS index, fracture consolidation time, and operative time between the two groups. For the authors, both the Krackow suture combined with the bridge suture technique and the tension band technique with Kirschner wire could achieve comparable clinical efficacy in the stable fixation of IPPF, subsequently allowing for the early initiation of rehabilitation exercises, while the bridge suture technique could also reduce the incidence of complications, hospital costs, and the need for surgical reoperation.

The analysis of the articles demonstrated that there was a general agreement among the authors on the need for stable fixation that would allow for early rehabilitation and knee movement to optimize clinical outcomes. Many studies, such as that of He et al.^8^ with the edge plate, Ma et al.^15^ with the Hand Plate System (HPS), and Pu et al.^14^ with external immobilization without tension, highlighted satisfactory results in terms of bone consolidation and recovery of ROM with their respective techniques. The low rate of clinical failure and good functional recovery are points of convergence, with some studies, such as those by Liu et al.^16^ and Zhou et al.^17^ reinforcing the idea that the stability of fixation is crucial for these positive outcomes, using methods such as tension band combined with suture anchors and Krackow suture with bridge suture.

However, there are notable disagreements and variations in technique preferences and associated complications. For example, while Chang and colleagues,^5^ compared TBW and TOR, highlighting that implant removal was significantly more common in the TBW group and that low patella occurred immediately after TOR, Yan and colleagues (22) suggested that Krackow suture combined with the bridge suture technique could reduce the incidence of complications and the need for reoperation compared to the tension band technique with Kirschner wire. This latter technique, while effective, has been associated with a higher rate of reoperation and complications in some contexts. Wang et al.^9^ on the other hand, pointed out that NiTi PC outperformed the tension band with cerclage in terms of knee function scores, suggesting that some approaches could offer superior functional outcomes. Additionally, the occurrence of low patella, as observed by Murase et al.^21^, was a complication present in different techniques, although the authors did not find significant differences in clinical outcomes between patients with and without low patella. The choice of technique, therefore, seemed to depend on the surgeon's experience, the characteristics of the fracture, and the minimization of specific complications, such as the need for implant removal or the occurrence of low patella, with some authors, such as Lu et al.^10^ proposing new approaches, such as fixation with "fishing net" suture, which demonstrated greater stability in biomechanical studies and promising clinical outcomes.

## CONCLUSION

In summary, the studies converge in the search for stable fixation techniques for IPPF that promote bone consolidation and early functional recovery, although the approaches vary in their efficacy and complication profile. In this context, the choice of the ideal technique still depends on factors such as the type of IPPF, the surgeon's experience, and the minimization of adverse outcomes, such as the need for reoperation or the occurrence of low patella.

## Data Availability

Data will be made available upon request.
